# Heterologous production of corosolic acid, a phyto-insulin, in agroinfiltrated *Nicotiana benthamiana* leaves

**DOI:** 10.5511/plantbiotechnology.24.0420a

**Published:** 2024-09-25

**Authors:** Jutapat Romsuk, Pisanee Srisawat, Jekson Robertlee, Shuhei Yasumoto, Kenji Miura, Toshiya Muranaka, Hikaru Seki

**Affiliations:** 1 Department of Biotechnology, Graduate School of Engineering, Osaka University, Suita, Osaka 565-0871, Japan; 2 Industrial Biotechnology Initiative Division, Institute for Open and Transdisciplinary Research Initiatives, Osaka University, Suita, Osaka 565-0871, Japan; 3 Faculty of Life and Environmental Sciences, University of Tsukuba, Tsukuba, Ibaraki 305-8572, Japan

**Keywords:** corosolic acid, *Nicotiana benthamiana*, triterpenoids, Tsukuba system, ursolic acid

## Abstract

Triterpenoids, a group of specialized plant metabolites with substantial structural diversity, are promising for healthcare applications. Ursolic acid, a pentacyclic triterpenoid with therapeutic potential, is also important as a precursor of corosolic acid, which is known as a “phyto-insulin” for its insulin-like properties. Ursolic acid is synthesized from a linear 30-carbon precursor 2,3-oxidosqualene via cyclization to produce triterpene scaffold α-amyrin, followed by a series of oxidation steps at the C-28 position mediated by cytochrome P450 monooxygenases (CYPs) in the CYP716A subfamily. The Tsukuba system was developed for the high-level transient expression of foreign proteins in plant cells based on the use of a binary vector equipped with geminiviral replication system and a double terminator. In this study, we used the Tsukuba system to produce ursolic acid in *Nicotiana benthamiana* leaves via transient pathway reconstruction. We used an oxidosqualene cyclase identified from the medicinal legume *Bauhinia forficata*, exhibiting a preponderant α-amyrin-producing activity. Wild-type *Medicago truncatula* CYP716A12 and its mutants were assessed in terms of ursolic acid production. We improved the performance of MtCYP716A12 by co-expressing it with the appropriate cytochrome P450 reductase (CPR) isozyme as an electron-transfer partner and tested different *Agrobacterium* infiltration ratios to optimize the CPR : CYP ratio to maximize ursolic acid production. We also achieved high yield of corosolic acid by co-expressing *Avicennia marina* CYP716C53 with ursolic acid biosynthetic enzymes. Moreover, engineering of AmCYP716C53 significantly improved corosolic acid yield, resulting in a yield exceeding the content found in banaba leaves, a well-known rich source of corosolic acid.

## Introduction

Triterpenoids are a group of specialized plant metabolites with substantial structural diversity and therapeutic potential (Yadav et al. 2010; Yan et al. 2014). Corosolic acid (2α-hydroxy-ursolic acid), a pentacyclic triterpenoid contained in the mature leaves of *Lagerstroemia speciosa* (commonly referred to as Banaba) is well known as a “phyto-insulin” and has recently attracted attention for its insulin-like properties, but without induction of anti-insulin antibodies (Jayakumar et al. 2014; Yang et al. 2016). Corosolic acid decreases blood glucose levels and has antihyperlipidemic and antioxidant effects, which are mediated by increased cellular glucose uptake, impaired sucrose and starch hydrolysis, diminished gluconeogenesis, and modulation of lipid metabolism. Consequently, banaba extract and corosolic acid can alleviate the symptoms of metabolic syndrome and confer other health benefits (Jayakumar et al. 2014; Miura et al. 2012; Schultz et al. 2018; Ulbricht et al. 2007).

Ursolic acid, a precursor of corosolic acid, is contained in numerous plant species, including many food, aromatic and medicinal plants (Gudoityte et al. 2021). Ursolic acid is often found in epicuticular waxes to prevent water loss or serve as a defense barrier against pathogens (for a review, see Gudoityte et al. 2021). Ursolic acid has also many pharmaceutical properties (for a review, see Woźniak et al. 2015). Particular attention has been given to the application of ursolic acid as an anticancer agent. The structural versatility of ursolic acid also renders it useful for the synthesis of derivatives, thereby facilitating the development of novel therapeutics (Gudoityte et al. 2021; Liu HR et al. 2021).

**Figure 1:**
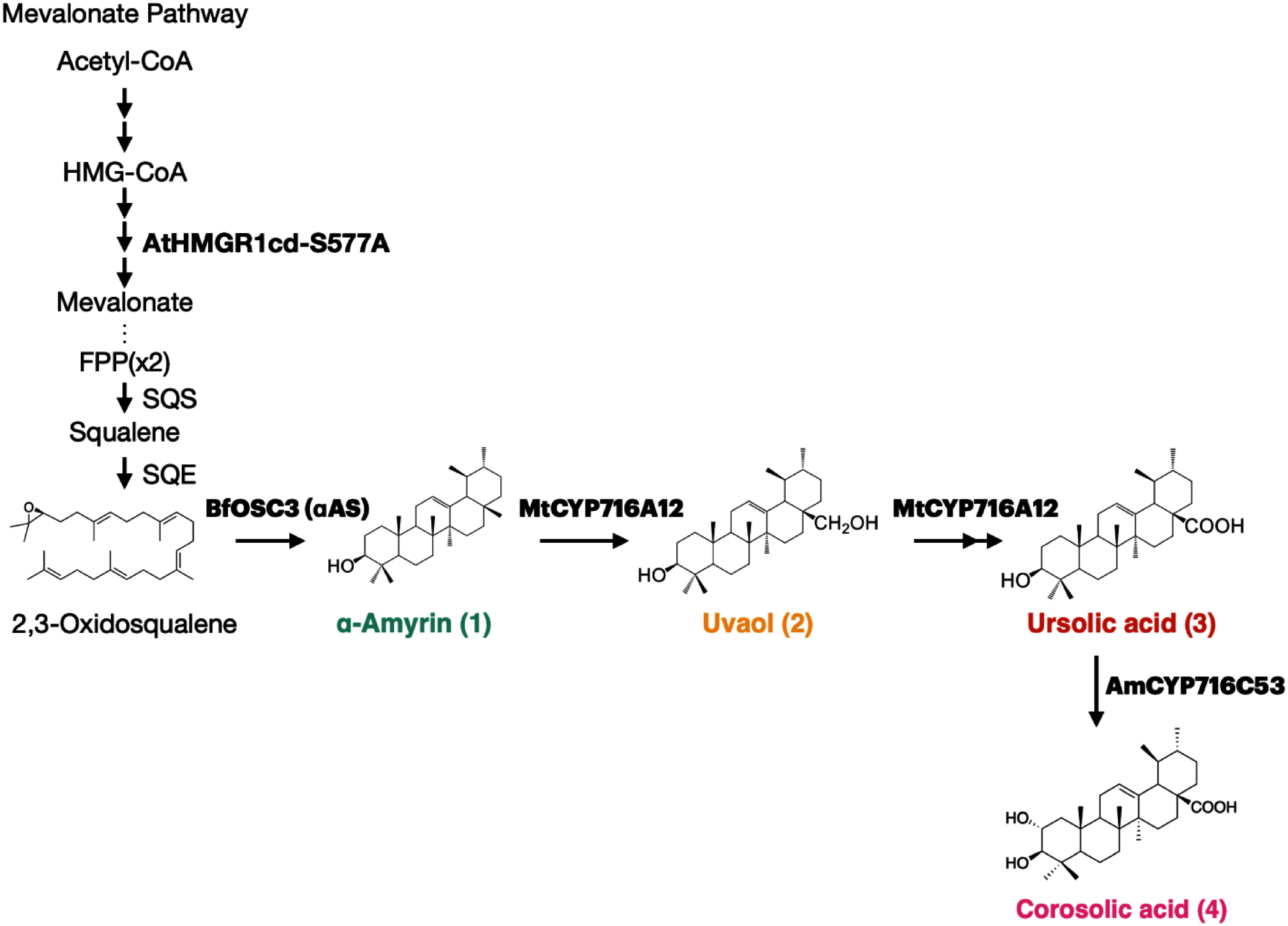
Figure 1. Biosynthetic pathway for bioactive triterpenoids involving catalysis by CYP716A12 and CYP716C53 enzymes transiently expressed in *N. benthamiana* leaves. An arrow between 2,3-oxidosqualene and α-amyrin (1) indicates a cyclization reaction catalyzed by an oxidosqualene cyclase (OSC). Single and double arrows downstream of α-amyrin (1) indicate one and two oxidation steps, respectively. FPP, farnesyl pyrophosphate; SQS, squalene synthase; SQE, squalene epoxidase; αAS, α-amyrin synthase; CYP, cytochrome P450 monooxygenase.

Triterpenoids are biosynthesized from 2,3-oxidosqualene, a common C30 acyclic precursor produced through the mevalonate pathway, via the cyclization by a group of enzymes called oxidosqualene cyclases (OSCs). Subsequently, the cyclic triterpene backbone undergoes site-specific oxidation by cytochrome P450 monooxygenases (CYPs) to produce triterpenoids with various structures (Seki et al. 2015). The biosynthesis of corosolic acid involves the cyclization of 2,3-oxidosqualene by an OSC, i.e., α-amyrin synthase (αAS), leading to the production of α-amyrin (1). Subsequently, α-amyrin undergoes sequential three-step oxidation at the C-28 position, producing ursolic acid (3) as a direct precursor for corosolic acid (4), through uvaol (28-hydroxy-α-amyrin) (2) and ursolic aldehyde as reaction intermediates (Figure 1). This oxidative reaction is primarily performed by CYP716A subfamily (Fukushima et al. 2011; Suzuki et al. 2018). The CYP716A subfamily enzymes, exemplified by *Medicago truncatula* CYP716A12 which is the first functionally characterized enzyme belonging to the CYP716A subfamily (Carelli et al. 2011; Fukushima et al. 2011), show a high level of functional preservation for triterpenoid biosynthesis, i.e. catalyzing three-step oxidation at the C-28 position as a common alteration to the different triterpene backbones, α-amyrin, β-amyrin, and lupeol (Miettinen et al. 2017). The final step in the biosynthesis of corosolic acid is C-2α hydroxylation of ursolic acid catalyzed by CYP716C subfamily enzyme (Dai et al. 2019; Miettinen et al. 2017; Nakamura et al. 2018).

Banaba extracts have been used for many years as traditional medicine to treat diabetes. The antidiabetic property of banaba is attributed to corosolic acid (Jayakumar et al. 2014; Stohs et al. 2012). However, Jayakumar et al., reported significant variations in the percentage distribution of corosolic acid, ranging from 0.005% to 0.868% DW among banaba populations (Jayakumar et al. 2014). Moreover, the accumulation of corosolic acid in raw materials depends on the plant age. In addition, as described in detail in the discussion section, corosolic acid was a minor oxidized triterpenoid product in our previous attempts to produce corosolic acid in engineered yeast de novo (Nakamura et al. 2018). These observations have motivated us to establish a method for heterologous production of corosolic acid using different production hosts.

“Tsukuba system” is the agroinfiltration-based transient protein expression system, which enables high-level transient expression of foreign proteins in plant cells, using the binary vector containing geminiviral replication system and a double terminator to boost expression (Yamamoto et al. 2018). Our previous study demonstrated that the Tsukuba system is suitable for the high-yield production of bioactive triterpenoids, oleanolic acid, and maslinic acid by heterologous expression of their biosynthetic enzymes in *Nicotiana benthamiana* (Romsuk et al. 2022a).

In this study, we applied the Tsukuba system to produce ursolic and corosolic acids in *N. benthamiana* by pathway reconstruction and engineering of CYP enzymes. Initially, we assessed the potential of wild-type MtCYP716A12 and its mutant enzymes as α-amyrin C-28 oxidase for high-yield ursolic acid production in *N. benthamiana* leaves. An optimal interaction between cytochrome P450 reductase (CPR) and CYP is essential for guaranteeing CYP functionality. Therefore, we improved the activity of MtCYP716A12 by co-expressing it with an appropriate CPR isozyme as an electron transfer partner. We also assessed the optimal ratio of *Agrobacterium* strains harboring different enzyme genes in infiltration mixture to optimize CPR : CYP ratio and maximize ursolic acid production. Finally, a ursolic acid production platform was used to produce corosolic acid (4). Co-expression of the ursolic acid biosynthesis gene sets with engineered AmCYP716C53, encoding an enzyme that catalyzes the C-2α-hydroxylation of ursolic acid (Nakamura et al. 2018), led to high-yield production of corosolic acid in agroinfiltrated *N. benthamiana* leaves at higher accumulation levels than in banaba leaves, which have been known as a rich source of corosolic acid.

In conclusion, we succeeded in rapidly producing substantial quantities of ursolic acid and its downstream compounds in agro-infiltrated *N. benthamiana* leaves, which is difficult when using conventional techniques.

## Materials and methods

### Authentic chemical standards

The triterpenoid standards used for metabolite analysis by gas chromatography-mass spectrometry (GC-MS)—α-amyrin (1), uvaol (2), ursolic acid (3), corosolic acid (4), and erythrodiol—were purchased from Extrasynthase (Lyon, France).

### Plasmid construction

The binary vector pYS_015 (Srisawat et al. 2019), a gateway-compatible version of the pRI 201-AN vector (TaKaRa Bio, Shiga, Japan), was used to evaluate AtHMGR1S and its derivatives. We used the gateway-compatible version of pBYR2HS (Romsuk et al. 2022a; Yamamoto et al. 2018) for the high-yield production of ursolic acid and corosolic acid using the Tsukuba system. The entry clones for MtCYP716A12 and its mutants, OeCYP716A48, AmCYP716C53, *Arabidopsis thaliana* 3-hydroxy-3-methylglutaryl-CoA reductase (HMGR1) and its derivatives, *Medicago truncatula* NADPH-cytochrome P450 reductases (CPRs), MtCPR1 and MtCPR2, and *Lotus japonicus* CPRs, LjCPR1 and LjCPR2, were obtained in previous studies (Fukushima et al. 2011; Istiandari et al. 2021; Nakamura et al. 2018; Robertlee et al. 2018; Seki et al. 2008; Suzuki et al. 2018). Generation of AmCYP716C53 mutant (CYP716C53_C210S) was conducted by site-directed mutagenesis using the PrimeSTAR® Mutagenesis Basal Kit (TaKaRa Bio, Shiga, Japan) and primers (Supplementary Methods 1). A codon-optimized gene for *Bauhinia forficata* α-amyrin synthase BfOSC3 (Srisawat et al. 2019) for expression in *N. benthamiana* was designed by using GeneOptimizer™ (GeneArt GmbH, Regensburg, Germany) and synthesized. The coding sequences (CDSs) of target genes were transferred to gateway-compatible versions of pBYR2HS or pYS_015 using Gateway LR Clonase II Enzyme Mix (Thermo Fisher Scientific, Waltham, MA, USA) to generate expression clones.

### Transient expression in *N. benthamiana* leaves

Transformation of *A. tumefaciens* strain GV3101 (pMP90) with expression constructs and selection of transformants was performed as previously described (Romsuk et al. 2022a). *Agrobacterium* suspensions for agroinfiltration were prepared as previously described (Romsuk et al. 2022a; Yamamoto et al. 2018). *Agrobacterium* suspension with the desired constructs was combined and infiltrated into the lower leaf surfaces of 5-week-old *N. benthamiana* plants using a needleless 1-ml syringe. To prevent tissue necrosis, the ascorbic acid solution was sprayed onto leaves 1, 3, and 5 days after infiltration, as described (Nosaki et al. 2021; Romsuk et al. 2022a). The leaves of the three plants were collected for triterpenoid analysis 7 days after infiltration.

### Homology modeling

Three-dimensional models of MtCYP716A12, *Centella asiatica* CYP716C11, and AmCYP716C53 (amino acid sequences provided in Supplementary Methods 2) were constructed using homology modeling. This process initiated with a Protein BLAST (BlastP) (Madden et al. 1996) search using a BLOSUM62 matrix against the Protein Data Bank (PDB) database (Burley et al. 2023) to identify analogous sequences within the protein structure database. The model exhibiting the highest degree of homology to the experimental data was selected as the template. Subsequently, the sequences of the target proteins were aligned with the template sequence. Model construction, refinement, and validation were executed utilizing Modeller 10.4 software (Webb and Sali 2017). Notably, the homology models included heteroatoms (non-water HETATM residues) and underwent optimization with a multi-domain assembler. Visualization of the models was accomplished using Chimera 1.17 (Pettersen et al. 2004), and the model integrity was verified using VERIFY3D software, confirming that more than 80% of the amino acids achieved a minimum score of 0.2 in the 3D/1D profile, thereby corroborating the efficacy of the homology modeling process (Eisenberg et al. 1997).

### Molecular docking analysis

Molecular docking analysis was performed to examine the interactions between AmCYP716C53 and ursolic acid and to identify the most effective binding. The chemical structures of the ligands were sourced from the Cambridge Structural Database (https://www.ccdc.cam.ac.uk/theccdcprofile/ (Accessed Jul 31, 2024)) and the PubChem database (https://pubchem.ncbi.nlm.nih.gov (Accessed Jul 31, 2024)). We used the docking score to predict the binding affinities. The I-TASSER modeling pipeline predicted the ligand-binding site for docking (Yang et al. 2015), which was executed using AutoDock Vina 1.1.2 via Chimera 1.17 (Pettersen et al. 2004). Our parameters included a box center at 50×5×15 and dimensions of 30×30×30 along the x-, y-, and z-axes, respectively. Selection criteria for binding poses encompassed the lowest docking score, the minimal distance between C-2α hydroxylation sites on ursolic acid backbones, and proximity from the heme reaction center to the ferrous iron (Fe^2+^) moiety (Geisler et al. 2013; Romsuk et al. 2022b; Yuki et al. 2012). Catalytic site capacity, amino acid side-chain position and conformation, ligand orientation, and ligand position were selected as docking evaluation criteria (docking profile). Structural alignment and homology modeling were performed using the MtCYP716A12 structure as a reference. The ClustalX algorithm (Thompson et al. 2003) was used to align and match MtCYP716A12, CaCYP716C11, and AmCYP716C53 during homology modeling. A residue-residue cut-off distance of 5 Å was set, aligning a residue in a column if it met the criteria with at least one other residue. The results were visualized using Chimera 1.17 (Pettersen et al. 2004).

### In silico site-directed mutagenesis

Candidate residues for site-directed mutagenesis were identified using PyMOL 2.5.5 software (Schrodinger, 2015). The native target residue in the AmCYP716C53 homology model was then replaced with a different amino acid. A hydrogen molecule was added and the homology model was retrained to maintain hydrogen bonding. The structure was exported in PDB format. Protein structures were optimized and visualized using Chimera 1.17 (Pettersen et al. 2004). Finally, structure quality was assessed using VERIFY3D (Eisenberg et al. 1997).

### Extraction of metabolites from *N. benthamiana* leaves

Metabolites were extracted from *N. benthamiana* leaves as previously described, with slight modifications (Romsuk et al. 2022a). To quantify triterpenoids in *N. benthamiana* leaves, 20 µl of erythrodiol (100 ppm in methanol) was added to 10 mg of lyophilized leaf samples. After extraction with 1 ml methanol, the samples were vortexed, sonicated for 60 min at 45% power using a bath sonicator (UT-206, SHARP, Osaka, Japan), and centrifuged at 12,000 rpm for 5 min. The organic phase was isolated and evaporated and the residue was reconstituted in methanol. The saponification involved methanol and 4 M HCl, heating at 80°C for 1 h, and incubation at room temperature. After adding ethyl acetate and hexane (1 : 1 v/v), and centrifugation at 1,500 rpm for 3 min, the organic phase was isolated, evaporated, and reconstituted in 500 µl methanol. The prepared samples were then stored at 4°C.

### Analysis of triterpenoids in leaf extracts by GC-MS

Triterpenoids in the leaf extracts were analyzed as previously described with slight modifications (Romsuk et al. 2022a, b). To assess triterpenoid levels in *N. benthamiana*, 50 µl of the sample underwent centrifugal drying, followed by derivatization with *N*-methyl-*N*-(trimethylsilyl) trifluoroacetamide at 80°C for 30 min. Concurrently, identical methods were applied to 50-µl standard solutions of α-amyrin (1), uvaol (2), ursolic acid (3), corosolic acid (4), and erythrodiol at 10 ppm. Gas chromatography-mass spectrometry (GC-MS) was performed using a 5977A MSD and 7890B Gas chromatograph equipped with an HP-5MS capillary column. The oven temperature started at 80°C for 1 min, increased to 300°C at 20°C min^−1^, and was held for 18 min. Helium was used as a carrier gas at a flow rate of 1 ml min^−1^. Mass spectra ranged from 50 to 750 *m*/*z*, and retention times and spectra were compared with those of the standards. Triterpenoid concentrations in *N. benthamiana* leaves were quantified using standard curves of the mentioned compounds (Supplementary Figure S1).

### Statistical analysis

To evaluate variations in triterpenoid concentrations, we performed a one-way analysis of variance (ANOVA). The significance of the differences in the means was evaluated using Tukey’s test. Between-group comparisons of oxidized triterpenoid levels were performed using unpaired Student’s *t*-tests. Values of *p*<0.05 were considered statistically significant. Statistical analyses were performed using JASP 0.16 for macOS (JASP Team, University of Amsterdam, Netherlands).

## Results

### Efficacy of the S577A mutation in AtHMGR1 for the production of α-amyrin in *N. benthamiana* leaves

To produce ursolic acid from a native 2,3-oxidosqualene pool in *N. benthamiana* leaves, we decided to use α-amyrin synthase (BfOSC3) identified from the medicinal legume tree *B. forficata* (Srisawat et al. 2019). We previously identified four OSCs linked to a range of triterpenoids in *B. forficata*. Among them, only BfOSC3 exhibited a preponderant α-amyrin-producing activity, which accounted for at least 95% of the total reaction products (Srisawat et al. 2019).

It is well known that HMGR is a rate-limiting enzyme of the mevalonate pathway. Therefore, we used AtHMGR1 to enhance the mevalonate pathway flux. Our previous study showed that introducing the S577A mutation into the N-terminal-truncated AtHMGR1, which encodes the catalytic domain (AtHMGR1cd, amino acid residues 166–592), improved its activity in vitro and made it into a negative feedback-insensitive form by avoiding phosphorylation (Robertlee et al. 2017, 2018). Moreover, the co-expression of AtHMGR1cd bearing the S577A mutation (AtHMGR1cd-S577A) with other biosynthetic enzymes significantly enhanced heterologous triterpenoid production in transiently transformed *N. benthamiana* leaves (Romsuk et al. 2022a). However, in our previous study, we did not compare the efficacy of AtHMGR1cd and AtHMGR1cd-S577A in transiently transformed *N. benthamiana*. Therefore, we compared the efficacy of AtHMGR1cd and AtHMGR1cd-S577A co-expressed with BfOSC3 in *N. benthamiana* leaves. As shown in the Supplementary Figure S2, AtHMGR1cd-S577A provided a more significant increase in α-amyrin yield (4.46±0.30 mg g^−1^ dw) compared to AtHMGR1cd (2.55±0.31 mg g^−1^ dw). A positive effect of S577A mutation on α-amyrin yield was also observed for full-length AtHMGR1 (Supplementary Figure S2).

However, because we used a conventional binary vector in this experiment, the yield of α-amyrin was much lower than when we used the expression vector of the Tsukuba system (pBYR2HS), which is equipped with geminiviral replication machinery, as described later.

### Wild-type CYP716A12 showed the highest potential for use in the production of ursolic acid in *N. benthamiana*

**Figure 2:**
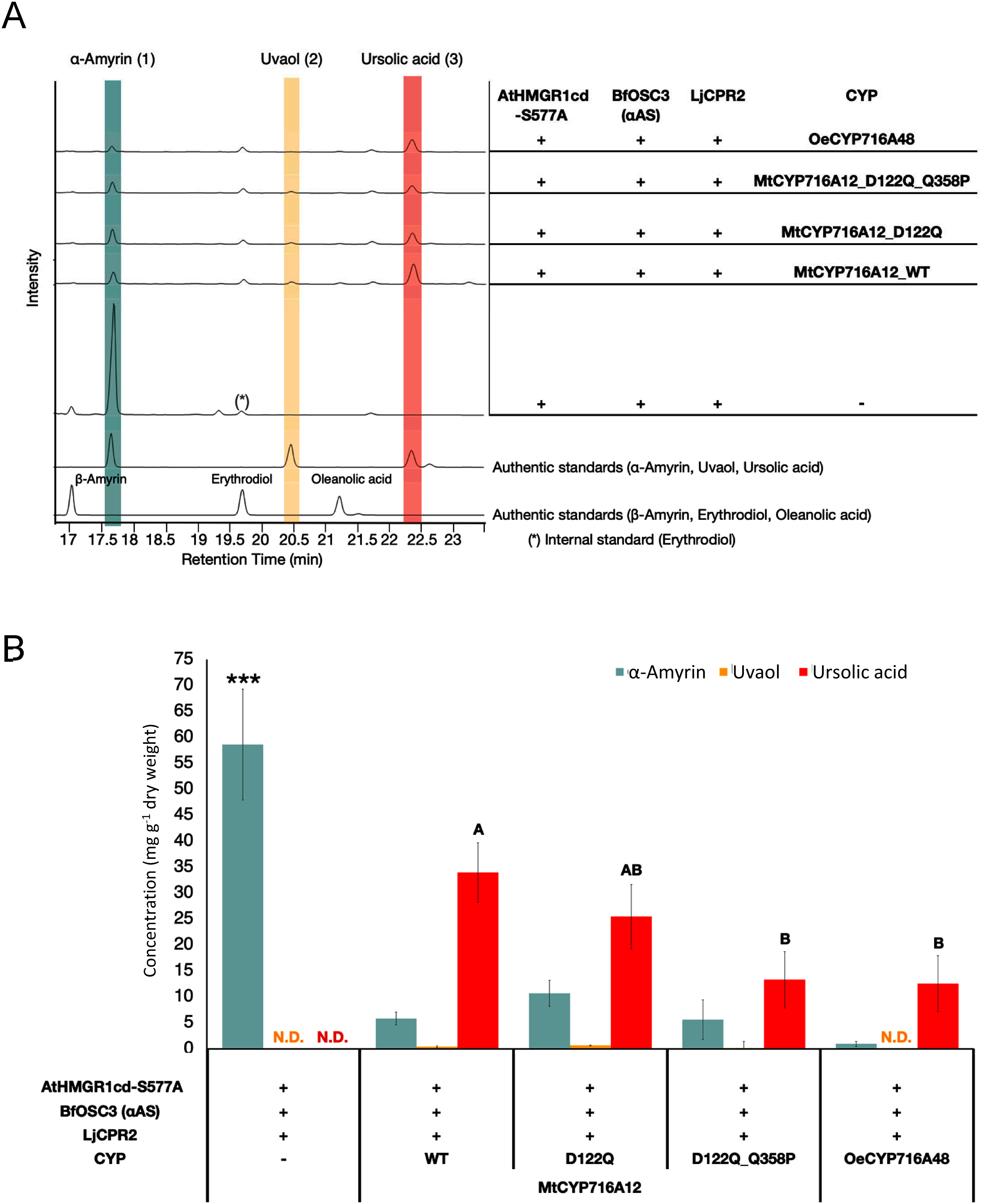
Figure 2. Comparison of CYP716As for ursolic acid production in *N. benthamiana.* (A) Total ion chromatograms (TIC) of extracts of *N. benthamiana* leaves transiently co-expressing AtHMGR1cd-S577A (AtHMGR), BfOSC3 (αAS, α-amyrin synthase), LjCPR2, and CYP716A (CYP716A48, CYP716A12_D122Q, CYP716A12_D122Q_Q358P, or wild-type CYP716A12) or empty vector as a control (-). Mixtures of α-amyrin (1), uvaol (2) and ursolic acid (3), and β-amyrin, erythrodiol and oleanolic acid were used as authentic standards; erythrodiol (*) was used as the internal standard. (B) Triterpene concentrations in the *N. benthamiana* leaf extracts analyzed in (A). Values and error bars are means and standard deviations. Data are representative of at least three biological replicates from three independent experiments (*n*=3). Letters indicate significant differences in ursolic acid levels (a–b) among samples (one-way ANOVA with Tukey’s post-hoc test, *** *p*<0.005). N.D. not detected.

To establish a high-yield production platform for ursolic acid, we reconstructed expression clones of BfOSC3 and AtHMGR1cd-S577A using the Tsukuba system expression vector, pBYR2HS. Leaves co-expressing AtHMGR1cd-S577A and BfOSC3 using pBYR2HS were harvested at 7 days after agroinfiltration and subjected to GC-MS analysis, resulting in a substantial accumulation of α-amyrin (1) as major product in *N. benthamiana* leaves at 58.60±10.69 mg g^−1^ dry weight (Figure 2, Supplementary Figure S3).

We also compared the abilities of different CYP716As, that is, wild-type MtCYP716A12, its mutant enzymes, and OeCYP716A48, using pBYR2HS. We have previously identified amino acid residues that influence the catalytic activity and substrate specificity of MtCYP716A12. Replacement of key residues in the substrate recognition sites (SRSs) of MtCYP716A12 alters its triterpenoid product profile. Two mutant MtCYP716A12s, D122Q and D122Q_Q358P, showed enhanced production of ursolic acid when expressed in *Saccharomyces cerevisiae* strain engineered to produce α-amyrin in vivo (Romsuk et al. 2022b). We also tested OeCYP716A48 identified from olive (*Olea europaea*), because OeCYP716A48 gave a higher ursolic acid production yield than MtCYP716A12 when expressed in an α-amyrin-producing yeast (Suzuki et al. 2018).

Equal volumes of *A. tumefaciens* harboring pBYR2HS expressing AtHMGR1cd-S577A, BfOSC3, LjCPR2, or each of the candidate CYP716As were mixed and infiltrated into *N. benthamiana* leaves. A mixture of *A. tumefaciens* harboring AtHMGR1cd-S577A, BfOSC3, LjCPR2, and an empty vector was used as a background control. The triterpenoid profile of leaf extracts were analyzed by GC-MS. The metabolite profiles of *N. benthamiana* leaves transiently expressing all the genes in the presence of CYP, revealed a prominent peak corresponding to an oxidized triterpenoid. This major peak was identified as ursolic acid (3) based on a comparison with an authentic standard (Figure 2A, Supplementary Figure S3). The triterpenoid concentrations in the leaf extracts were investigated (Figure 2B). The highest concentration of ursolic acid (3) was detected in the leaves expressing wild-type MtCYP716A12 (33.92±5.75 mg g^−1^ dw) (Figure 2B).

Therefore, MtCYP716A12 showed the highest potential for ursolic acid production, and it was selected for further experiments.

### Enhancing the performance of CYPs using the *M. truncatula* CPR

CYPs require electrons from the CPR for their oxidative activity. However, when CYPs are introduced into a heterologous host, the host CPRs are not always suitable partners (Liu T et al. 2021; Qu et al. 2015; Xu et al. 2004). To address this issue, CPR expression can be increased or alternative electron transfer partners can be used (Istiandari et al. 2021; Liu T et al. 2021; Theron et al. 2019). Our previous study demonstrated that the co-expression of an additional CPR in the Tsukuba system can improve the performance of heterologous CYPs in *N. benthamiana*; for example, it substantially increased oleanolic acid production (Romsuk et al. 2022a).

Plants have multiple CPR genes that branch into two classes, class I and class II (Istiandari et al. 2021; Qu et al. 2015), which have been proposed to be responsible for primary and specialized metabolism, respectively (Parage et al. 2016; Qu et al. 2015). To identify optimal CYP : CPR pairs, we co-expressed wild-type MtCYP716A12 with each of the four CPRs belonging to class I (LjCPR1 and MtCPR1) or class II (LjCPR2 and MtCPR2).

**Figure 3:**
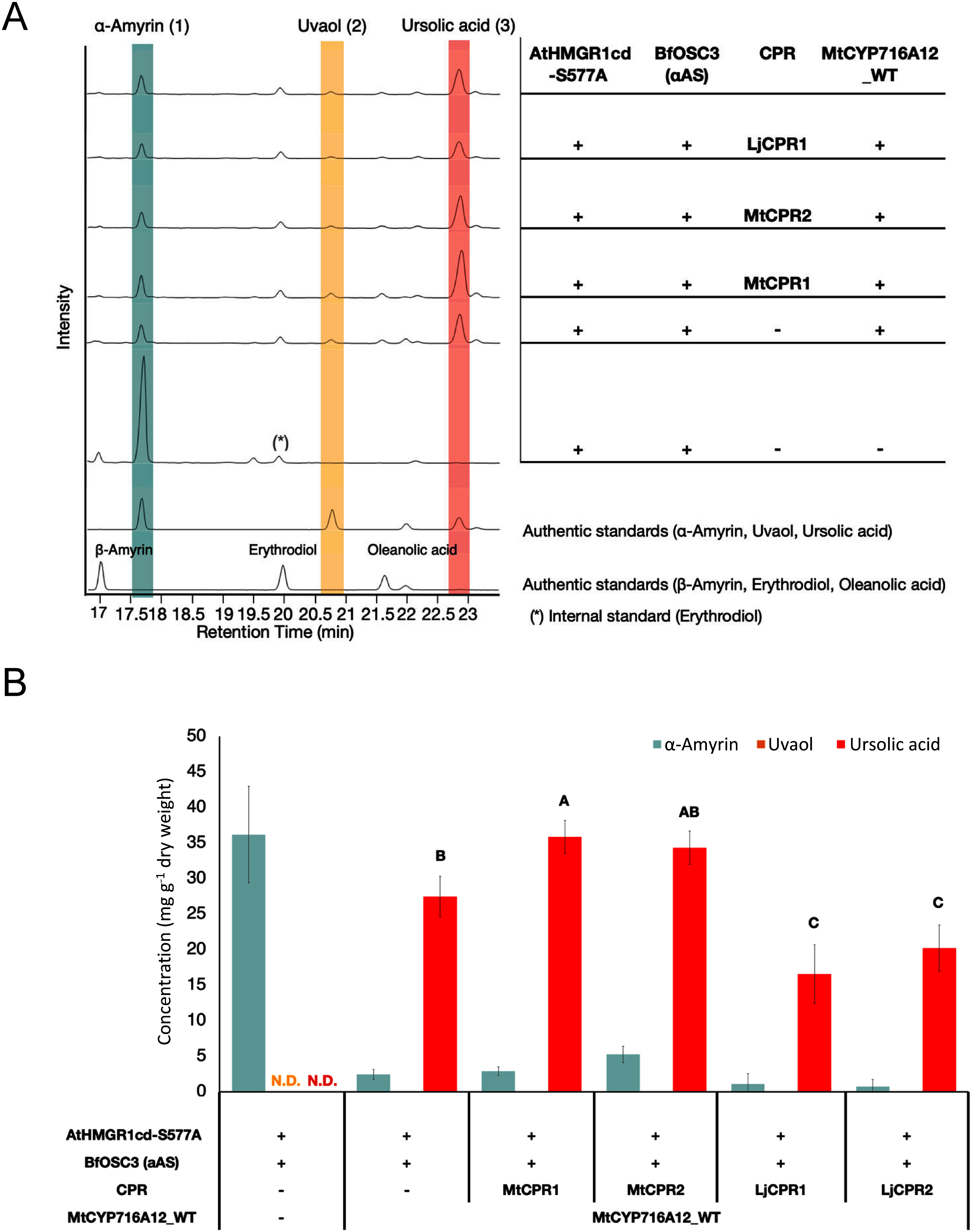
Figure 3. Optimal CPR-CYP pairing enhances ursolic acid production in *N. benthamiana*. (A) TICs of extracts of *N. benthamiana* leaves co-expressing AtHMGR1cd-S577A (AtHMGR), BfOSC3 (αAS, α-amyrin synthase), wild-type CYP716A12, and each of different CPR. Two control experiments were also conducted, i.e., lacking both CPR and CYP716A12, or lacking CPR only. Mixtures of α-amyrin (1), uvaol (2) and ursolic acid (3), and β-amyrin, erythrodiol and oleanolic acid were used as authentic standards; erythrodiol (*) was used as the internal standard. (B) Triterpene concentrations in the *N. benthamiana* leaf extracts analyzed in (A). Values and error bars are means and standard deviations. Data are representative of at least three biological replicates from three independent experiments (*n*=3). Letters indicate significant differences in ursolic acid levels (a–b) among samples (one-way ANOVA with Tukey’s post-hoc test). N.D. not detected.

To evaluate the efficacy of the additional CPR, equal volumes of *A. tumefaciens* harboring pBYR2HS expressing AtHMGR1cd-S577A, BfOSC3, wild-type MtCYP716A12, and each of the candidate CPRs were mixed and infiltrated. Two control experiments were conducted, lacking both additional CPR and MtCYP716A12, or lacking additional CPR only (Figure 3). Quantification of ursolic acid in the leaf extracts were performed by GC-MS. In all the co-infiltrated samples, excluding the background control lacking MtCYP716A12, a prominent peak corresponding to ursolic acid and a minor peak corresponding to uvaol were observed (Figure 3A), as determined by comparison with the corresponding authentic standard. Triterpenoid concentrations were investigated, as shown in Figure 3B. When MtCYP716A12 was co-expressed with CPR originating from *M. truncatula*, MtCPR1, or MtCPR2, ursolic acid yield was increased to 35.87±2.32 and 34.35±2.35 mg g^−1^ dw, respectively, compared to the control lacking additional CPR only (27.46±2.88 mg g^−1^ dw) (Figure 3B). In contrast, when MtCYP716A12 was co-expressed with LjCPR1 or LjCPR2, the accumulation of ursolic acid was lower than that without additional CPR, indicating that a suitable pair of CYP and CPR is needed to improve the accumulation of ursolic acid in *N. benthamiana* leaves.

Consequently, the level of ursolic acid was high in the leaves co-expressing CPR originating from the same species (*M. truncatula*) as MtCYP716A12, and no obvious differences were observed between class I and class II CPRs. Based on these results, MtCPR1 was selected as an additional electron-transfer partner of MtCYP716A12 for further experiments.

### Effect of the ratio of *Agrobacterium* strains harboring biosynthetic genes on triterpenoid production

Optimization of *Agrobacterium* suspension ratio for co-infiltration of multiple strains with unique expression constructs is important for the reconstitution of multistep metabolic pathways (Carlson et al. 2023). We hypothesized that the ratio of *Agrobacterium* strains in the co-infiltration solution, particularly the CPR : CYP ratio, would significantly affect ursolic acid yield.

**Figure 4:**
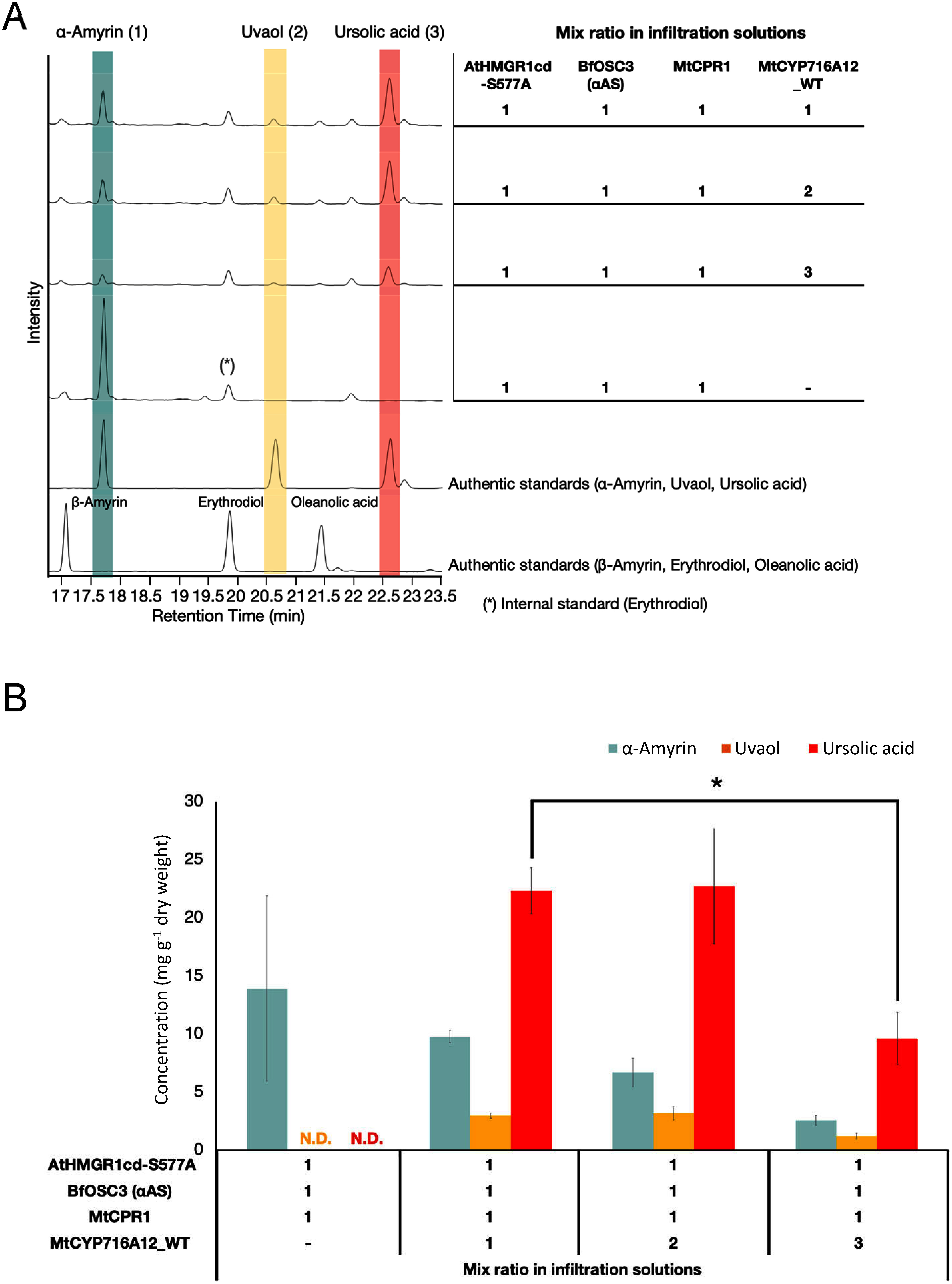
Figure 4. Enhancement of ursolic acid production by optimizing the *Agrobacterium* infiltration ratio. (A) TICs of extracts of *N. benthamiana* leaves co-infiltrated with a mixture of *Agrobacterium* strains harboring AtHMGR1cd-S577A (AtHMGR), BfOSC3 (αAS, α-amyrin synthase), MtCPR1, and wild-type CYP716A12, at the indicated ratios keeping total infiltration OD_600_ at 1.0. Infiltration without wild-type CYP716A12 was conducted as a background control (-). Mixtures of α-amyrin (1), uvaol (2) and ursolic acid (3), and β-amyrin, erythrodiol and oleanolic acid were used as authentic standards; erythrodiol (*) was used as the internal standard. (B) Triterpene concentrations in the *N. benthamiana* leaf extracts analyzed in (A). Values and error bars are means and standard deviations. Data are representative of at least three biological replicates from three independent experiments (*n*=3). The significance of differences in ursolic acid levels among samples was evaluated by one-way ANOVA with Tukey’s post-hoc test (* *p*<0.05). N.D. not detected.

To validate this hypothesis, different *Agrobacterium* ratios were assessed to determine the ursolic acid yield in *N. benthamiana*. In all co-infiltrated samples, except for the control sample lacking CYP716A12, a prominent peak corresponding to ursolic acid (3) was observed, as determined by comparison with the corresponding authentic standard (Figure 4A). The concentration of ursolic acid in leaves was 22.35±1.98 mg g^−1^ dw with the co-infiltration ratio of AtHMGR1cd-S577A, BfOSC3, MtCPR1, and wild-type MtCYP716A12 was 1 : 1 : 1 : 1 (Figure 4B). Comparable yield (22.73±4.94 mg g^−1^ dw) was obtained with the ratio of AtHMGR1cd-S577A, BfOSC3, MtCPR1, and wild-type MtCYP716A12 was 1 : 1 : 1 : 2 (Figure 4B). Importantly, the ursolic acid yield was significantly decreased (9.61±2.26 mg g^−1^ dw) when the ratio of AtHMGR1cd-S577A, BfOSC3, MtCPR1, and wild-type MtCYP716A12 was 1 : 1 : 1 : 3 (Figure 4B). These results show that the ratio of *Agrobacterium* strains, each containing a gene for ursolic acid biosynthesis, co-infiltrated into *N. benthamiana* affects ursolic acid yield. Based on the results shown in Figure 4, an equal proportion of *Agrobacterium* was used to produce corosolic acid.

### Production of corosolic acid in *N. benthamiana* leaves and yield improvement via the engineering of AmCYP716C53

Corosolic acid is a derivative of ursolic acid (2α-hydroxy-ursolic acid), contained in banaba leaves. Our previous study identified AmCYP716C53 from gray mangrove (*Avicennia marina*) and demonstrated its ability to catalyze the C-2α hydroxylation of ursolic acid to produce corosolic acid when expressed in ursolic acid-producing yeast (Nakamura et al. 2018). However, among the oxidized triterpenes detected in yeast, corosolic acid was detected as a minor product (Nakamura et al. 2018). We also successfully enhanced the catalytic activity of AtCYP716A1 and AtCYP716A2 through protein engineering, based on the results of a comparative functional analysis of MtCYP716A12 and its variants (Romsuk et al. 2022b). Based on these findings, we focused on the substrate recognition site (SRS) II, that was predicted as previously described by Gotoh (1992), particularly the residue situated within the enzyme’s binding pocket (residues 207–211), to enhance the activity of AmCYP716C53 through amino acid substitution. CaCYP716C11, identified from *Centella asiatica*, selectively catalyzes C-2α hydroxylation of pentacyclic triterpenoids, oleanolic acid and ursolic acid (Miettinen et al. 2017). Dai et al. (2019) also reported that, in engineered yeast, CaCYP716C11 (that was renamed in Dai et al. 2019 as CaCYP716C49 for unknown reasons, although CYP716C11 is the original CYP name authorized by the Cytochrome P450 nomenclature committee) facilitates the biosynthesis of 141 mg l^−1^ corosolic acid and shows a marked substrate preference for oleanolic acid and ursolic acids over betulinic acid (Dai et al. 2019).

**Figure 5:**
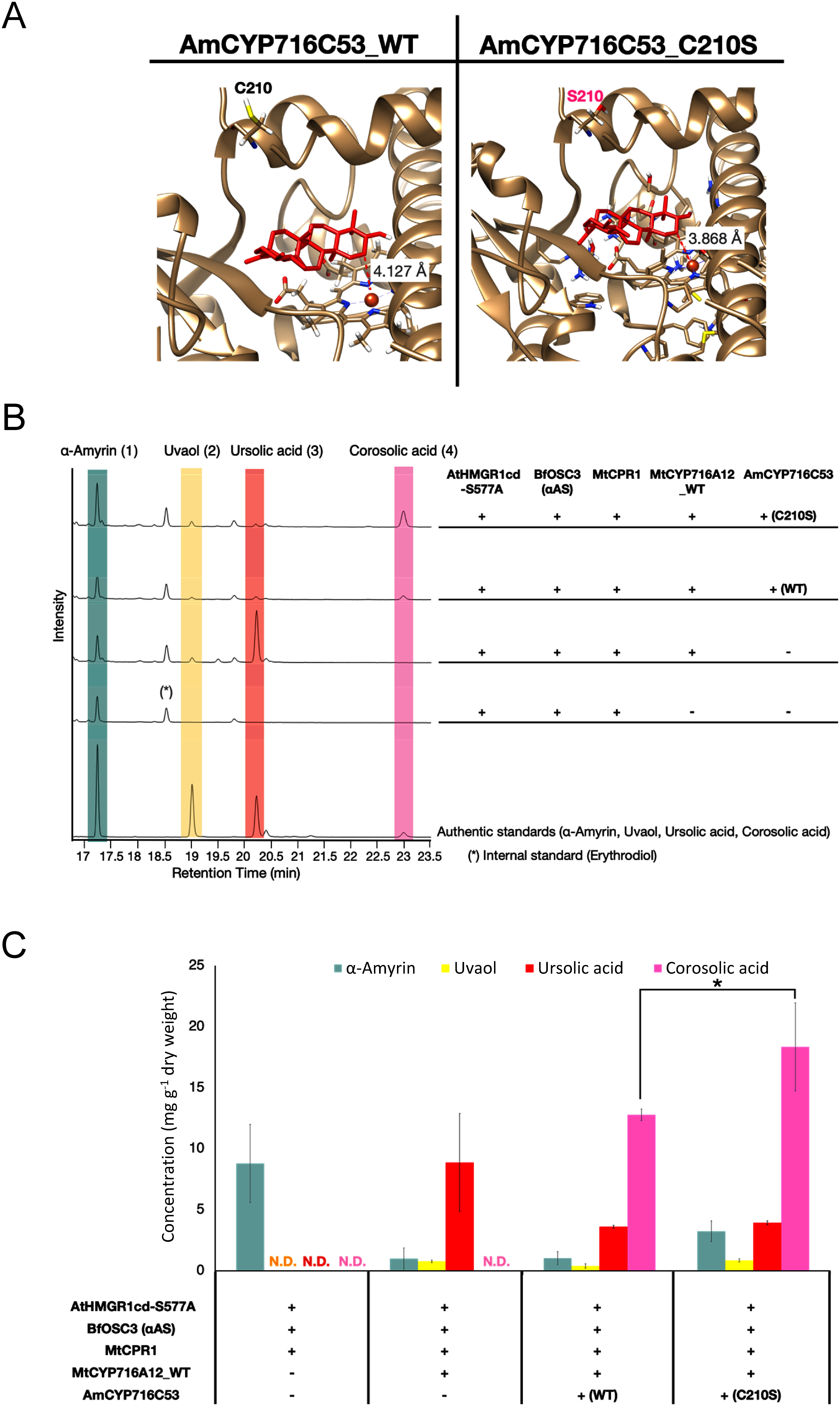
Figure 5. Corosolic acid production in *N. benthamiana* and yield improvement by engineering of CYP716C53. (A) Representative interactions of wild-type CYP716C53 and CYP716C53_C210S with the ursolic acid backbone. (B) TICs of extracts of *N. benthamiana* leaves co-expressing AtHMGR1cd-S577A (AtHMGR), BfOSC3 (αAS, α-amyrin synthase), MtCPR1, wild-type CYP716A12, and wild-type CYP716C53 or CYP716C53_C210S. Infiltration without CYP716C53 was conducted as control (-). α-Amyrin (1), uvaol (2), ursolic acid (3), and corosolic acid (4) were used as authentic standards; erythrodiol (*) was used as the internal standard. (C) Corosolic acid concentration in *N. benthamiana* leaf extracts. Data represent at least three biological replicates (*n*=3) and are shown as means±standard errors of the mean. Differences in corosolic acid concentrations were assessed using Student’s *t*-test (* *p*<0.05). N.D. not detected.

Structural alignment was performed by comparing the targeted amino acid residues (residues 207 to 211) within the SRSII region of both CaCYP716C11 and AmCYP716C53. We found a polymorphic site between the two CYP enzymes specifically at amino acid residue 210. In CaCYP716C11, this residue is serine (S), whereas in AmCYP716C53, it is cysteine (C) (Supplementary Figure S4D). Subsequently, we conducted in silico site-directed mutagenesis by substituting the cysteine at position 210 with serine in AmCYP716C53 (resulting in AmCYP716C53_C210S) (Figure 5A). This modification altered the docking profile of AmCYP716C53 with ursolic acid. The orientation of the substrate, ursolic acid, within the binding pocket of both the wild-type (AmCYP716C53) and mutant (AmCYP716C53_C210S) enzymes was found to be nearly identical. However, when considering the direction and distance from the target site (C-2), the mutant enzyme displayed closer alignment with the heme-Fe^2+^ reaction center than the wild-type enzyme, implying a more centralized positioning (Figure 5A). Based on these results, we hypothesized that this alteration may affect the catalytic activity, potentially influencing the production of corosolic acid in *N. benthamiana*.

To produce corosolic acid, we infiltrated the leaves with a mixture of *A. tumefaciens* harboring pBYR2HS expressing AtHMGR1cd-S577A, BfOSC3, MtCPR1, wild-type MtCYP716A12, wild-type AmCYP716C53, or AmCYP716C53_C210S. As shown in Figure 5B, a peak corresponding to corosolic acid (4), confirmed by comparison with an authentic standard (Supplementary Figure S3), was detected when AmCYP716C53 or AmCYP716C53_C210S was co-expressed. Corosolic acid was quantified to compare the yields of wild-type AmCYP716C53 and AmCYP716C53_C210S (Figure 5C). The yield of corosolic acid significantly increased by 1.44-fold upon co-expression of AmCYP716C53_C210S (18.36±3.61 mg g^−1^ dw) compared with wild-type AmCYP716C53 (12.79±0.48 mg g^−1^ dw) (Figure 5C).

## Discussion

In this study, we showed that the simultaneous expression of AtHMGRcd-S577A, BfOSC3, and wild-type MtCYP716A12 in *N. benthamiana* using the Tsukuba system can produce ursolic acid in high yields. Indeed, the concentration of ursolic acid in leaves (33.92±5.75 mg g^−1^ dw) (Figure 2B) was 1.65-fold higher than the concentration in flowers of *Calendula officinalis* (20.5 mg g^−1^ dw) which was the highest concentration among 23 plant species reviewed in Gudoityte et al. (2021). In the present study, the wild-type MtCYP716A12 (Figure 2B) showed the highest ursolic acid yield when expressed in *N. benthamiana*. Conversely, when expressed in yeast, the wild-type OeCYP716A48 showed the highest ursolic acid yield, followed by MtCYP716A12_D122Q_Q358P. The inconsistent results could be attributed to the use of different expression systems, which influence the catalytic activity and yield of oxidized triterpenoids (Czarnotta et al. 2017; Romsuk et al. 2022a).

CYP activity is dependent on electron transfer from CPR (Mellor et al. 2019). In heterologous hosts, compatibility may be compromised due to physiological and genetic differences. Therefore, it is necessary to optimize CPR expression and identify alternative partners (Istiandari et al. 2021; Liu T et al. 2021; Theron et al. 2019). Unlike mammals, which have one CPR gene, plants have multiple CPR genes that branch into class I and class II (Istiandari et al. 2021; Qu et al. 2015). Class I and class II CPR have been proposed to be involved in primary and specialized metabolism, respectively (Parage et al. 2016; Qu et al. 2015). In engineered yeast, maximal CYP716A activity was achieved by the co-expression of CPR belonging to class I from the same species as CYP, resulting in higher triterpenoid production (Istiandari et al. 2021). In our previous study, we co-expressed an additional class II CPR (LjCPR2) using the Tsukuba system to maximize MtCYP716A12 activity in *N. benthamiana*, which resulted in increased oleanolic acid production (Romsuk et al. 2022a). In the present study, we compared class I (MtCPR1 and LjCPR1) and class II (MtCPR2 and LjCPR2) CPRs for MtCYP716A12 activity during ursolic acid production in *N. benthamiana*. Wild-type MtCYP716A12 provided the highest ursolic acid yield when co-expressed with MtCPR1, followed by MtCPR2, LjCPR2, and LjCPR1 (Figure 3), indicating that there was no obvious correlation with different CPR classes and that it is still difficult to rationally determine the optimal partner. Therefore, co-expression of additional CPR originating from the same species as the heterologous CYPs may be a reasonable strategy because they should have co-evolved in vivo. Optimization of the CPR : CYP ratio is also important for maximizing CYPs functionality. Indeed, changing *Agrobacterium* ratio for the co-infiltration of biosynthesis genes into *N. benthamiana* influenced the ursolic acid yield (Figure 4). CYPs are usually abundant beyond CPR in plant cells, since excess expression of CPR is toxic for the cells by causing electron transfer uncoupling and the generation of reactive oxygen species (Renault et al. 2014). However, unexpectedly, excess CYP (the ratio of CPR and CYP was 1 : 3) in the co-infiltration assay led to a reduction in ursolic acid yield (Figure 4). Although the reasons why an excess of CYP in the co-infiltration assay led to a reduction in ursolic acid yield are still unclear, it would be possible that the decrease in the proportion of AtHMGR1cd-S577A and BfOSC3 in the total with the increase in the proportion of CYP could have resulted in a decrease of α-amyrin, a substrate for CYP to produce ursolic acid. Indeed, when the ratio of AtHMGR1cd-S577A, BfOSC3, MtCPR1, and wild-type MtCYP716A12 was 1 : 1 : 1 : 3, the accumulation of α-amyrin was lower than that with the ratio of 1 : 1 : 1 : 2 or 1 : 1 : 1 : 1 (Figure 4B).

Corosolic acid is a triterpenoid abundant in banaba leaves that can enhance blood sugar regulation and aid in the management of diabetes (Jayakumar et al. 2014; Miura et al. 2012; Schultz et al. 2018; Ulbricht et al. 2007). When AmCYP716C53 was co-expressed with α-amyrin synthase, LjCPR2, and AmCYP716A259 (α-amyrin C-28 oxidase from *A. marina*) in yeast, corosolic acid was a minor product, because a substantial amount of α-amyrin and ursolic acid remained to be consumed by AmCYP716C53 to produce corosolic acid (Nakamura et al. 2018). By contrast, corosolic acid was the major product when AmCYP716C53 was expressed in *N. benthamiana* leaves (Figure 5). We also engineered the AmCYP716C53 mutant by replacing a critical amino acid in the SRSII domain to generate AmCYP716C53_C210S. This approach led to a corosolic acid yield of 18.36±3.61 mg g^−1^ dw which is a 1.44-fold increase compared with wild-type AmCYP716C53 (Figure 5C). This concentration is 4.32 times higher than the reported mean level found in matured banaba leaves (4.25 mg g^−1^ dw) (Jayakumar et al. 2014). Jayakumar et al. (2014) also reported significant variations in the percentage distribution of corosolic acid, ranging from 0.005% to 0.868% DW among populations, and the accumulation of corosolic acid in raw plant materials may depend on plant age. Indeed, the highest concentration of corosolic acid has been detected in mature leaves of banaba (Jayakumar et al. 2014). Therefore, it should also be noted that the yield of 18.36±3.61 mg g^−1^ dw was obtained at 7 days after agroinfiltration.

In summary, this study achieved a high yield heterologous production of ursolic acid, a pivotal bioactive triterpenoid, and corosolic acid, which is well known as a “phyto-insulin”, in *N. benthamiana* leaves by integrating the Tsukuba system and protein engineering. Our results introduce an alternative strategy to produce health-beneficial triterpenoids other than extraction from natural plant resources, which will also promote innovation in drug discovery.

## Description of Supplementary Files

Supplementary Methods.

Figure S1. Standard curves for quantification of (A) α-Amyrin, (B) Uvaol, (C) Ursolic acid and (D) Corosolic acid in *N. benthamiana* leaf extracts.

Figure S2. Advantages of AtHMGR1S and its variants in enhancing triterpenoid production in *N. benthamiana* leaves.

Figure S3. Mass fragmentation pattern of triterpenoids peaks from the GC profile.

Figure S4. Structural alignment of AmCYP716C53 with CaCYP716C11 and MtCYP716A12.
