# Supplementary material for: Heterologous production of corosolic acid, a phyto-insulin, in agroinfiltrated Nicotiana benthamiana leaves
Source: Plant Biotechnol (Tokyo). 2024 Sep 25;41(3):277–88. doi: 10.5511/plantbiotechnology.24.0420a (PMC11921146; doi:10.5511/plantbiotechnology.24.0420a)
Supplement: Supplementary Data [file plantbiotechnology-41-3-24.0420a-s001.pdf]

Supplementary Materials

Supplementary Methods

1. The specific primers for site-directed mutagenesis of AmCYP716C53.

Primer name	Forward (5’->3’)	Reverse (5’->3’)
**AmCYP716C53_C210S**	GGTATGCATTCTATCATGATT AATATTCCAGGG	AATCATGATAGAATGCATACC CACGGTCACATC

2. The protein sequence of the CYP716 family enzymes used in homology modeling.

>MtCYP716A12_*Medicago_truncatula* (Genbank accession no. ABC59076.1)
MEPNFYLSLLLLFVSFISLSLFFIFYKQKSPLNLPPGKMGYPIIGESLEFLSTGWKGHPEKFIFDRMRKYS
SELFKTSIVGESTVVCCGAASNKFLFSNENKLVTAWWPDSVNKIFPTTSLDSNLKEESIKMRKLLPQFFKP
EALQRYVGVMDVIAQRHFVTHWDNKNEITVYPLAKRYTFLLACRLFMSVEDENHVAKFSDPFQLIAAGIIS
LPIDLPGTPFNKAIKASNFIRKELIKIIKQRRIDLAEGTASPTQDILSHMLLTSDENGKSMNELNIADKIL
GLLIGGHDTASVACTFLVKYLGELPHIYDKVYQEQMEIAKSKPAGELLNWDDLKKMKYSWNVACEVMRLSP
PLQGGFREAITDFMFNGFSIPKGWKLYWSANSTHKNAECFPMPEKFDPTRFEGNGPAPYTFVPFGGGPRMC
PGKEYARLEILVFMHNLVKRFKWEKVIPDEKIIVDPFPIPAKDLPIRLYPHKA

>CaCYP716C11_*Centella_asiatica* (Genbank accession no. AOG74835.1)
MDLFLPLVFLSVILIVLIFKPRSDGDKKLPPGSFGWPIMGETIEFLFGHPKEFVDKRMKKYSPDIFKSNIL
GEKTAIICGPEGHKFLFSNEEKFFTVFRPHPIQRLFRSYNNKSAPDPPPSGAGSKDDVKSIKQPGFFKPEA
LSRFIGVIEATIQQHLRAHWEGKDTVEAYPLSKSLTLTLSCRFFLGIDNPERIARLVHMFDDITLGMHSII
SNVPGTVFYRAKNAAAAVRKELLCVIKEKKQEMAAGKKAQDVLSHMISFSDPSTGKFMPELEVADKMMGLI
TAGYSTVATSMAFLMKFVGESPAIYNKIRAEQIELAESKNPGEPLTWVDIQKLKYSWQAMCETMRLVPPLQ
GTFREVINEFTYAGYTVPKGWKVYWTVSTVHMNPKYFPNPEKFDPSRYEEGKISTPYTYVPFGGGPRMCPG
KEYARIAVLTFLHHVVRKYKWEVLFPDEKVIGDMMPAPEKGLPIRLHPH

>AmCYP716C53_*Avicennia_marina* (Genbank accession no. BBG80454.1)
MEFFAVAFSLALIALTVVFISRRRSDGGAKLPPGTFGWPIFGESIEFLFGKPEKFVGDRMKKYSPDIFKTK
VLGEKTAVICGPNGHKFLFSNEHKYFTAFRPHPMQHLFRSYKDKSAPPAETQRPDETKAIRQPGFLKPEAL
IRFLAKMDSITQQQLQVHCADKNEVEVYPLAKTVTLTLACQFFLGINNPERIARLVKYFDDVTVGMHCIMI
NIPGTIFYRANKAAAAIRKELVTVIREKKQAIASGSPMRDILSHMIVVTDPSGQSMPDYEIADKMMGLLTA
GYSTVATTITFLMKYVGLNPEVYERVRAEQLEIAASKKPDELLEWEDVGKMKYSWNVICETMRLVPPLQGT
FREVLTEFNYAGYTIPKGWKVYWTVSTTNTNPKYFKDSEKFNPSRYDEGEAPPPYTYVPFGGGPRMCPGKE
YARLSILTFVHNVVKRYKWEVIDPKEKVEGDMMPAPQKGLPIRLYHH

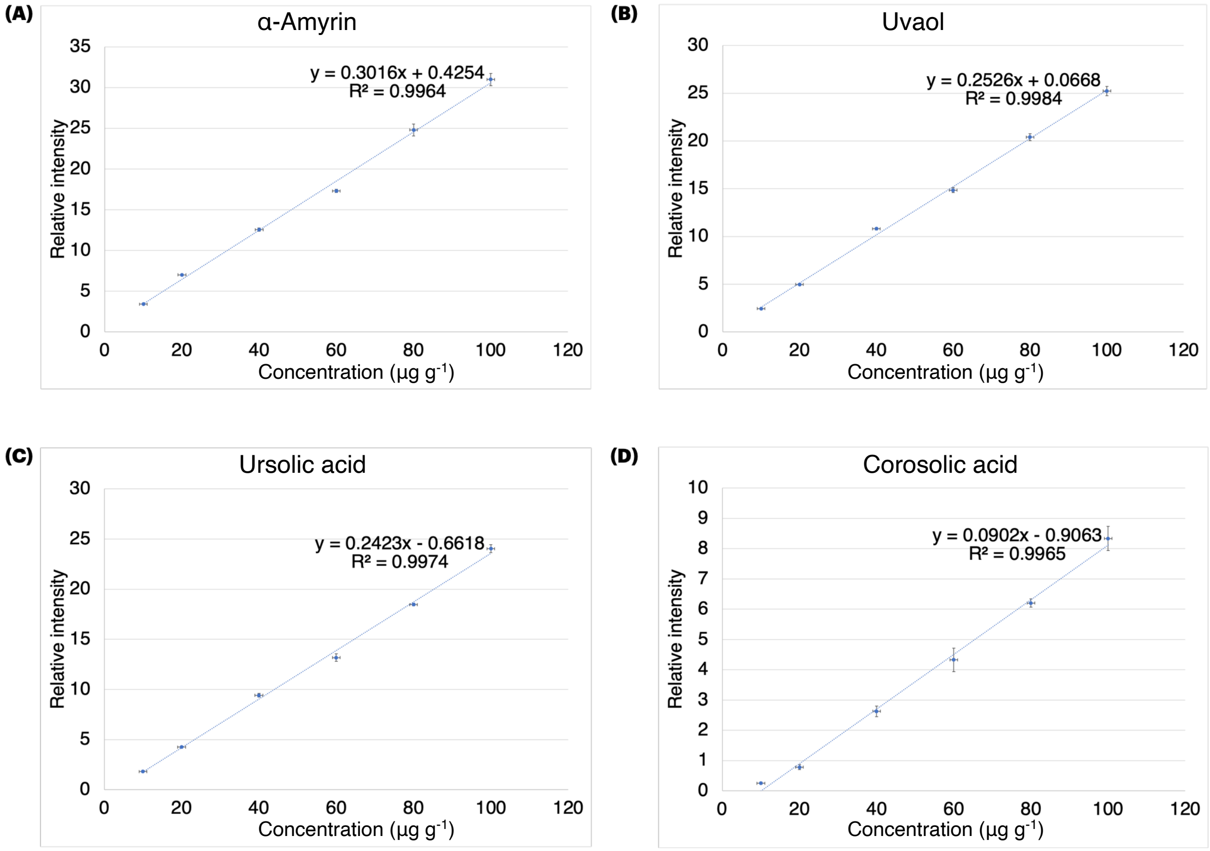

Supplementary Figure S1. Standard curves for quantification of (A) α-Amyrin , (B) Uvaol, (C) Ursolic acid and (D) Corosolic acid in *N. benthamiana* leaf extracts.
Standard curve, plot of peak areas versus concentrations of authentic standard compounds, was constructed. Mean values and standard deviation (error bars) for three replicates are shown.

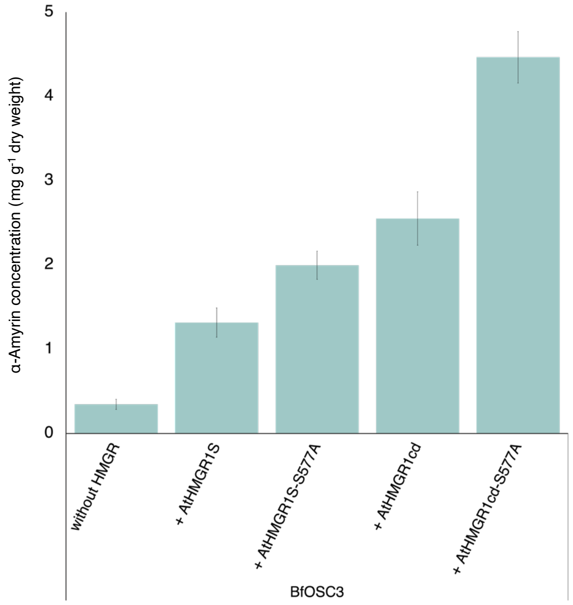

Strain	α-Amyrin (mg g^{-1} dry weight)	
	Average	SEM
BfOSC3/ without HMGR	0.3451	0.0604
BfOSC3/ AtHMGR1S	1.1315	0.1738
BfOSC3/ AtHMGR1S-S577A	1.9958	0.1678
BfOSC3/ AtHMGR1cd	2.5507	0.3171
BfOSC3/ AtHMGR1cd-S577A	4.4645	0.3059

Supplementary Figure S2. Advantages of AtHMGR1S and its variants in enhancing triterpenoid production in *N. benthamiana* leaves.

Quantification of α-amyrin was performed on extracts from *N. benthamiana* leaves co-expressing AtHMGR and BfOSC3, using a conventional binary vector pYS_015. Mean values and standard deviation (error bars) for five biological replicates are shown.

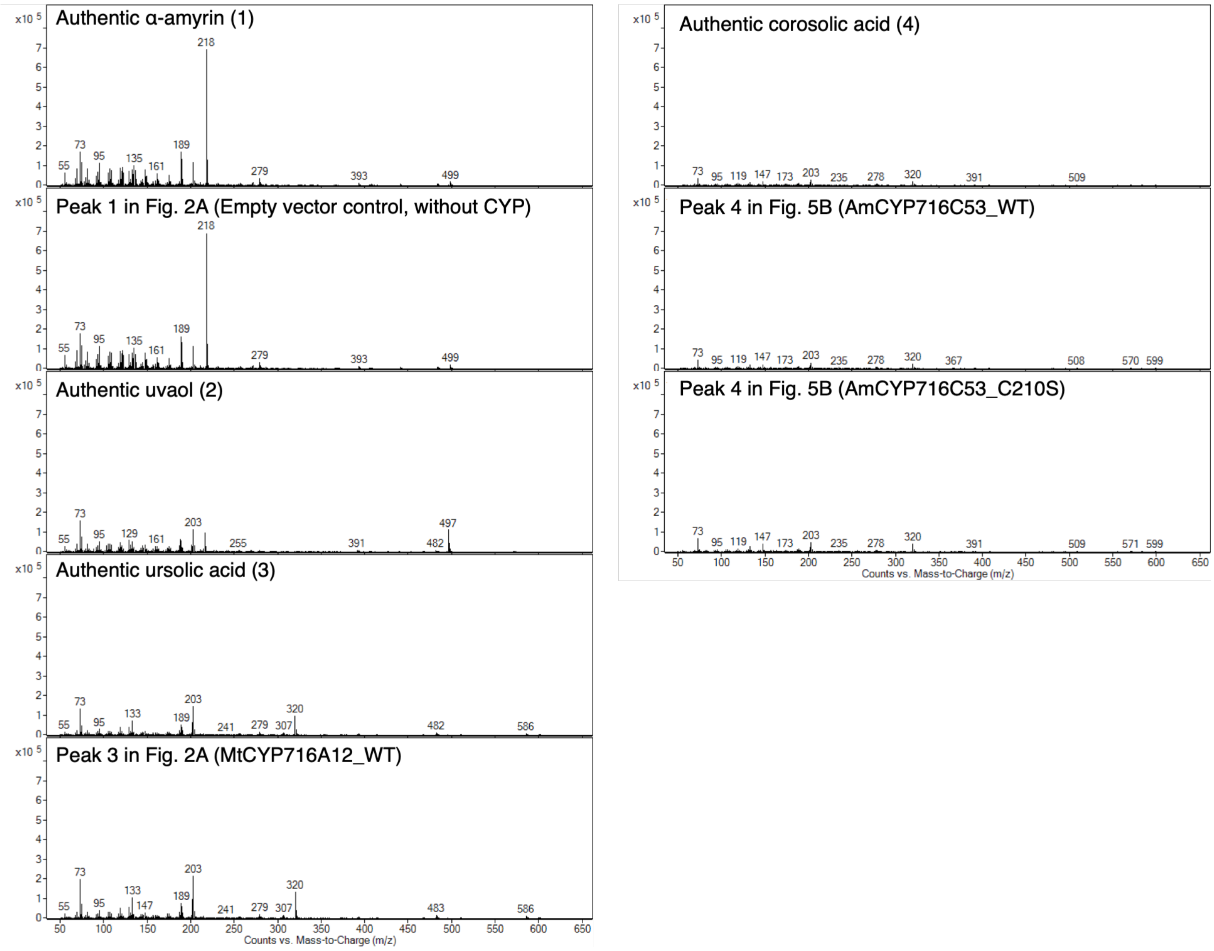

Supplementary Figure S3. Mass fragmentation pattern of triterpenoids peaks from the GC profile.

Mass spectra of peaks from the GC profile of *N. benthamiana* leaves extracts compared to those of authentic standard compounds.

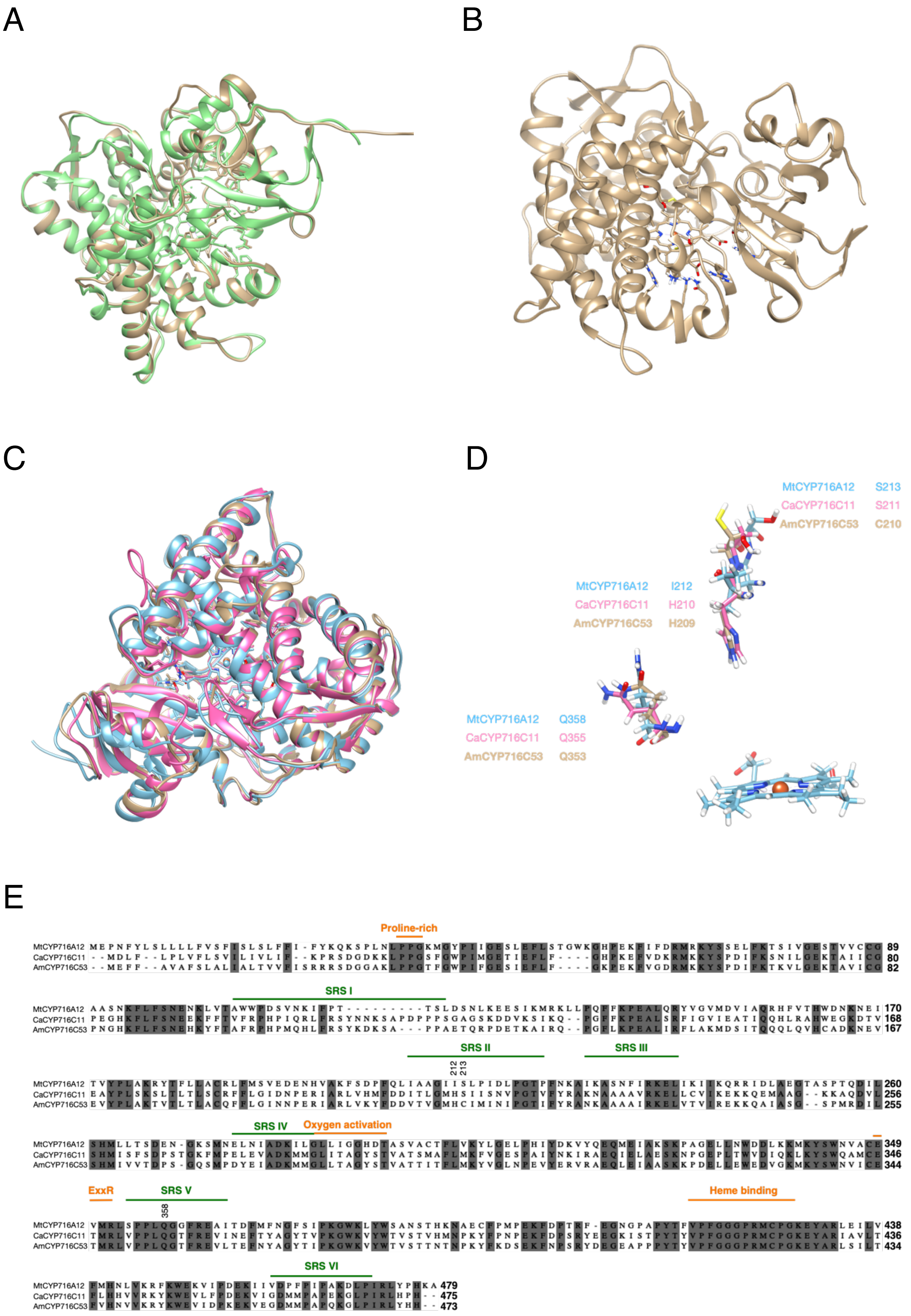
A
B
C
D
MtCYP716A12 S213
CaCYP716C11 S211
AmCYP716C53 C210
MtCYP716A12 I212
CaCYP716C11 H210
AmCYP716C53 H209
MtCYP716A12 Q358
CaCYP716C11 Q355
AmCYP716C53 Q353
E
Proline-rich
SRS I
SRS II
SRS III
SRS IV
Oxygen activation
ExxR
SRS V
Heme binding
SRS VI
MtCYP716A12
CaCYP716C11
AmCYP716C53
89
80
82
170
168
167
212
213
260
256
255
349
346
344
358
438
436
434
479
475
473

Supplementary Figure S4. Structural alignment of AmCYP716C53 with CaCYP716C11 and MtCYP716A12.

(A) The cyanobacterial CYP120A1 chain A, bound to retinoic acid (PDB ID: 2VE3_A), exhibits the closest resemblance to the experimental structure (33.6%). Consequently, it was chosen as the template model (Green), while the structure of AmCYP716C53 is represented in Tan. **(B)** The homology modeling of AmCYP716C53 surpassed the quality criteria established by VERIFY3D with an impressive score of 81.26%. **(C)** A structural alignment of AmCYP716C53 (Tan), CaCYP716C11 (Pink) and MtCYP716A12 (Blue). **(D)** Comparative analysis of the amino acid residues at the substrate recognition sites (SRS) II and IV by aligning the structures of AmCYP716C53 with those of CaCYP716C11 and MtCYP716A12. **(E)** Alignment of amino acid sequences of MtCYP716A12, CaCYP716C11, and AmCYP716C53, highlighting conserved motifs. Areas denoted for predicted substrate recognition sites (SRS), SRS I (106–125), SRS II (207–221), SRS III (227–237), SRS IV (276–286), SRS V (354–365), and SRS VI (460–469), predicted as previously described by Gotoh (1992), are colored in green. CYP signature motifs including the proline-rich motif (35–37), oxygen-binding motif (287–295), ExxR motif (349–352), and the heme-binding site (417–428) are depicted in orange.
